# Une complication rare de la grossesse gémellaire monochoriale: la séquence Twin-reversed arterial perfusion (TRAP)

**DOI:** 10.11604/pamj.2015.20.347.4197

**Published:** 2015-04-10

**Authors:** Sofia Jayi, Meriem Laadioui, Kamilia Laabadi, Fatima Zohra Fdili, Hakima Bouguern, Hikmat Chaara, Aabdelilah Melhouf

**Affiliations:** 1 Service de Gynécologie-Obstétrique 2, CHU Hassan II de Fès, Université Sidi Mohammed Benabdellah, Fes, Maroc

**Keywords:** Séquence TRAP, diagnostic prénatal, pronostic, TRAP, prenatal diagnosis, prognosis

## Abstract

La séquence TRAP est une forme majeure et rare du syndrome transfuseur transfusé, caractérisée par l'absence de développement des structures cardiaques avec un spectre de malformations chez le fœtus transfusé qui n'est jamais viable et d'importantes complications touchant le jumeau transfuseur. Nous rapportons le cas d'une multipare admise avec expulsion en cours d'une présentation de siège, puis l'examen a trouvé une présentation irrégulière. Et à l’échographie une image hétérogène sans aire cardiaque ni organes fœtaux individualisables avec une seule masse placentaire sont visible, évoquant en premier un jumeau acardiaque. La voie basse a été acceptée, mais elle a présenté une hypercinésie évoquant un syndrome de prérupture. La césarienne a permis l'extraction d'une masse acardiaque. A travers ce cas, nous insistons sur l'intérêt du diagnostic prénatal de cette entité dans l'adaptation de la prise en charge, l'amélioration du pronostic du jumeau transfuseur ainsi que l’évitement du retard diagnostic et de ces conséquences.

## Introduction

Le jumeau acardiaque, ou séquence TRAP (*twin-reversed arterial perfusion*), ou acardius acranius ou masse acardiaque est une pathologie sévère des grossesses gémellaires monochoriales [1, 2]. Il s'agit en effet d'une forme majeure et rare du syndrome transfuseur transfusé [3–6], caractérisée par l′absence de développement des structures cardiaques, associée à un spectre de malformations développementales et réductionnelles chez le fœtus appelé masse acardiaque [1]. Ce dernier n'est jamais viable et les complications touchant le jumeau transfuseur sont importantes [5]. Nous rapportons le cas d'une masse acardiaque, responsable d'un syndrome de prérupture utérine dans les suites de l'accouchement du premier jumeau, chez une jeune multipare dont la grossesse n'a pas été suivie.

## Patient et observation

**Figure 1:**
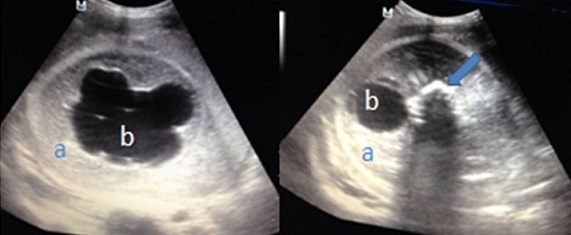
Images échographiques montrant la composante mixte: tissulaire (a), liquidienne (b) avec une structure osseuse (flèche)

**Figure 2:**
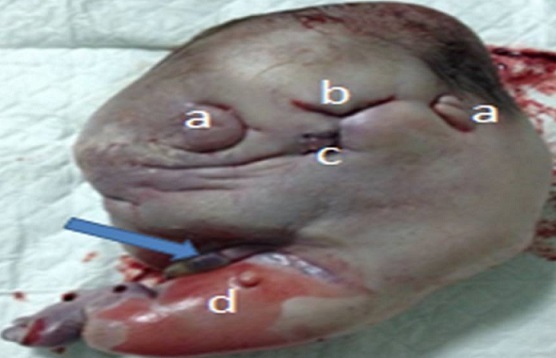
La masse acardiaque avec aspect tronqué du corps où manque la partie thoracique; ébauches d'oreilles (a), de nez (b), de la bouche (c), un membre inférieur malformé (d) et un cordon ombilical (flèche)

Madame M.K, 30 ans, 6^ème^ geste, 5^ème^ pare, grossesse non suivie, estimée à terme, admise pour accouchement. L'examen a trouvé une hauteur utérine à 33cm. Les contractions utérines présentent, les BCF sont perçus en un seul foyer, le toucher vaginal trouve un col à dilatation complète, une poche des eaux rompus une présentation de siège décomplété engagée en cours d'expulsion. La parturiente à évoluer rapidement vers l′accouchement par vois basse d′un nouveau né de sexe féminin, poids de naissance à 2900g, et l'apgar à 10/10. Puis le toucher vaginal à trouver une présentation très irrégulière d'où la réalisation de l’échographie à la salle de travail. Laquelle a objectivé une volumineuse formation ovale mesurant 16cm grand axe, hétérogène à contenu mixte- tissulaire (a) et liquidien (b) une structure osseuses (flèche) sans visualisation de l'aire cardiaque ni d'organes fœtaux individualisables (Figure 1). Par ailleurs, une seule masse placentaire fundique a été retrouvée, d’échostructure habituelle et complètement indépendante de la masse sus décrite. Le tout évoquant en premier un jumeau acardiaque. Ainsi, la voie basse a été acceptée, mais elle a présenté une hypercinésie avec syndrome de prérupture, d'où la décision de césarienne pour disproportion fœto-pelvienne. Laquelle a permis l'extraction d'un jumeau (Figure 2) pesant 1500g, ayant un corps tronquée où manque la partie thoracique, avec, ébauches d'oreilles (a), de nez (b) et de la bouche (c), quelques cheveux sont présent, un membre inférieur mal formé (d) et un cordon ombilical (flèche). L'examen fœto-pathologique a été refusé par la famille.

## Discussion

**Figure 3:**
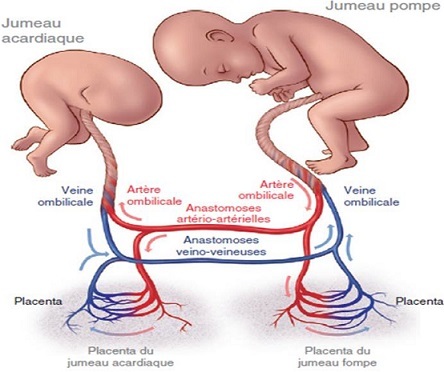
Schématisation de la circulation fœto-placentaire d'une grossesse avec un jumeau acardiaque. UV: veine ombilicale; UA: artère ombilicale; VV: veno-veineuse; AA: artério-artérielle

La présence de jumeau acardiaque est de survenue exceptionnelle il touche une naissance sur 35 000 [5, 6] et 1% des grossesses monozygotes [1]. Cette entité représente le second type de complications des anastomoses vasculaires des placentas monochoriaux après le syndrome transfuseur transfusé [5]. La pathogénie de cette complication rare est controversée [2], pour certains auteurs, le premium moyen serait une dysmorphogenèse cardiaque primaire et les anastomoses vasculaires placentaires ne seraient nécessaires que pour le développement du fœtus acardiaque. D'autres pensent que l'anomalie en cause serait la présence d'un flux vasculaire inversé responsable secondairement d'une atrophie cardiaque. En effet la théorie la plus privilégié actuellement est basée sur l'association de deux conditions obligatoires [7, 8] qui sont une insuffisance circulatoire de survenue précoce (entre 8 et 12 SA) chez le futur acardiaque et la présence d'anastomoses placentaires veno-veineuses et artério-artérielle [1, 2]. Le futur acardiaque peut être anatomiquement normal ou anormal. Il ressemble à un vrai parasite [7] qui est perfusé à partir du jumeau pompe par les artères ombilicales en un flux rétrograde [3] *via* des anastomoses de la plaque choriale artério-artérielles et venoveineuses (Figure 3) [3]. Ce sang étant pauvre en oxygène, entraine un défaut de développement de la tête, du cœur et des membres inférieures [7], d′où la dénomination de séquence *twin-reversed arterial perfusion* (TRAP) [1, 5]. Ce fœtus acardiaque n'est jamais viable et les complications concernant le fœtus pompe sont fréquentes à savoir, la prématurité, l'hydramnios, l'anasarque fœto-placentaire, la défaillance cardiaque (50% des cas) et la mort in utero qui survient dans 50% à 70% des cas [5].

En échographie, il se présente sous forme d'une masse d'allure tératomateuse, œdematiée avec des éléments anatomiques désorganisés, des zones anéchogènes de nature liquidienne (segment digestifs occlus) et échogènes (structures osseuses désorganisées). Le cordon ombilical est court et comporte très souvent une seule artère [3]. La visualisation d′une activité cardiaque n′exclut pas le diagnostic de la séquence TRAP [9]. Ce n′est que la visualisation d'un flux pulsatile inversé de l'artère ombilicale du fœtus acardiaque au Doppler couleur qui permettra de confirmer le diagnostic [1]. Par ailleurs, ce flux inversé ainsi que la croissance du jumeau acardiaque constituent des signes de vitalité en cas d'absence du cœur [2, 3]. L′étude du poids du jumeau acardiaque est difficile à réaliser en raison des malformations mais le développement de l’échographie 3D pourrait palier à ce problème en calculant le volume des fœtus par la méthode volumique [5].

Les diagnostics différentiels à évoquer sont la mort fœtale in utero d'un jumeau normal, anencéphale ou associé à un volumineux hygroma kystique; une tumeur placentaire ou intra-amniotique et également une grossesse biamniotique en assimilant la peau d'un acardiaque en anasarque comme la membrane inter-amniotique. La certitude du diagnostic doit se baser sur la constatation d'une perfusion rétrograde du jumeau acardiaque en mode Doppler couleur et pulsé [5, 10]. Cliniquement, la masse acardiaque se présente typiquement comme un corps tronqué auquel manque la partie supérieure thoraco-céphalique avec un ou deux membres inférieurs déformés avec un cœur absent ou rudimentaire [2].

Certains facteurs de mauvais pronostic ont été rapportés par les auteurs à savoir l'insuffisance tricuspidienne qui apparait plus précocement que les signes habituellement recherchés de l'insuffisance cardiaque imposant une surveillance échographique par un cardio-pédiatre [10]; une différence de l'indexe de pulsatilité (IP) entre les deux jumeaux même quand elle est faible, était associée à une issue de grossesse défavorable [5]; et Une inversion du flux du canal d'Arantius et une vitesse systolique de l'artère cérébrale élevée témoignant d'une anémie (due à un saignement dans la masse acardiaque [5, 10]. Par ailleurs, quand le rapport poids du fœtus acardiaque/ fœtus pompe dépasse 70%, le risque de prématurité est de 90%, l'hydramnios est observé dans 40% des cas et le risque de l'insuffisance cardiaque du jumeau-pompe est de 30% [3]. Certains caractères morphologiques ont été décrits comme étant associés à un haut risque de complications comme la présence d'un bras, la présence de reins ou d'une jambe [5].

**Figure 4:**
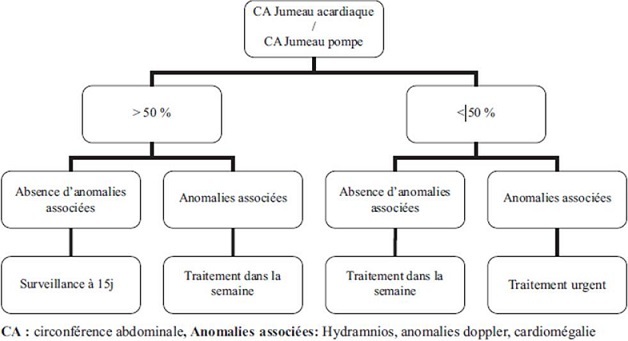
Critères de prises en charge en fonction des données échographiques

Plusieurs types de traitements prénataux peuvent être utilisés. Certains, sont à visée étiologique, visant à interrompre la circulation du fœtus acardiaque et ceci soit en traitant par laser les anastomoses entre les deux jumeaux, ou en utilisant la coagulation mono- ou bipolaire du cordon ombilical du jumeau acardiaque ou en embolisant ce cordon par des substances thrombogènes comme l'alcool [5] ou encore par destruction vasculaire percutanée par radiofréquence [3, 6]. Des traitements symptomatiques comme l'indométhacine (par son effet tocolytique et par la diminution du liquide amniotique), l'amniodrainage en cas d'hydramnios, ou encore la digitalisation peuvent être utilisés [5] Les attitudes thérapeutiques restent discutées, certains auteurs ont proposé une attitude conservatrice quand un ratio des IP des artères ombilicales des deux jumeaux inférieur à 1,3 ou une différence des index de résistance supérieure à 0,20 [5], alors que d'autres sont orientés essentiellement par le ratio circonférence abdominale du fœtus acardiaque/fœtus pompe (Figure 4) [1, 5]. Cependant, le traitement symptomatique est généralement indiqué en cas d'hydramnios majeur ou de défaillance cardiaque du jumeau pompe [3].

## Conclusion

La mortalité périnatale du jumeau pompe est de 35-50%, cependant, La précocité du diagnostic d'un jumeau acardiaque est cruciale pour une prise en charge optimale. Ainsi nous attirons l'attention des praticiens sur le grand intérêt du diagnostic prénatal précoce qui permettrait grâce à un suivi échographique régulier d'adapter la conduite à tenir selon les facteurs pronostic entre la simple surveillance et l'intervention anténatale et par conséquent d'améliorer le pronostic du jumeau pompe, et d’éviter les découvertes surprises de cette entité au cours du travail comme ça été le cas dans notre observation.
